# American Edition of Nothnagel’s Encyclopedia

**Published:** 1902-04

**Authors:** 


AMERICAN EDITION OF NOTHNAGEL'S ENCYCLOPEDIA.—Variola, vaccination, varicella, cholera, erysipelas, whooping cough, hay fever. Variola (including vaccination), by Dr. H. Immermann, of Basle. Varicella, by Dr. Th. von Jürgensen, of Tübingen. Cholera, Asiatic and Cholera Nostras, by Dr. C. Liebermeister, of Tübingen. Erysipelas and Erysipeloid, by Dr. H. Lenhartz, of Hamburg. Whooping Cough and Hay Fever, by Dr. G. Sticker, of Giessen. Edited, with additions, by Sir J. W. Moore, B. A., M. D., F. R. C. P. I., Professor of the Practice of Medicine, Royal College of Surgeons, Ireland. Handsome octavo volume of 682 pages, illustrated. Philadelphia and London: W. B. Saunders & Co. 1902. Cloth, $5.00 net; half Morocco, $6.00 net.

The articles contained in this volume of Nothnagel's Encyclopedia are, without exception, masterly expositions of the subjects treated in them. "Variola" and "Vaccination" are deserving of the highest praise. They are in every respect all that could be desired. Many of the interpolations and additions made by the editor add something to its value, but in some of the articles, notably, the article on "Whooping Cough," they seem to be far more numerous than necessary. Their multiplication would be less material if they were added as foot-notes instead of being scattered throughout the text, where they make frequent and often annoying interruptions. R.

